# Reconstruction of a Complex Pretibial Wound Using Dermal Regeneration Template and Split-Thickness Skin Graft

**DOI:** 10.7759/cureus.110360

**Published:** 2026-06-06

**Authors:** Abhya Niegocki, Caleb W Brown, Nicole M Ceausu, Brian Q Le, Jeremy M Powers

**Affiliations:** 1 Surgery, East Tennessee State University Quillen College of Medicine, Johnson City, USA; 2 General Surgery, East Tennessee State University Quillen College of Medicine, Johnson City, USA

**Keywords:** dermal matrix, exposed pretibial wound, negative pressure wound therapy, plastic and reconstructive surgery, staged reconstruction

## Abstract

The reconstruction of the distal leg is challenging due to watershed vascularity and scarce soft-tissue bulk. While traditional reconstruction guidelines rely on vascularized flap coverage, such interventions are higher-risk options in certain patients with significant medical co-morbidities. This case report describes non-microsurgical reconstruction of a distal-third pre-tibial wound in a patient with a history of renal transplant, type 2 diabetes, coronary artery disease, and peripheral vascular disease. In this patient, chronic immunosuppression induced blunt wound healing, while concurrent peripheral artery disease made microsurgery unreliable. We navigated this challenge by "engineering" a wound bed using a dermal regeneration template (DRT) in conjunction with antimicrobial negative pressure wound therapy (NPWT). Within four weeks, the wound showed robust granulation, with complete coverage of previously exposed tendon. The wound was then covered with a split-thickness skin graft (STSG) eight weeks after the initial application of DRT. The patient returned to his pre-injury functional status within five months. This staged strategy offers a lower-risk alternative to traditional flap-based protocols for patients with significant co-morbidities, including transplant immunosuppression and peripheral arterial disease.

## Introduction

The pretibial region is a challenging area for tissue remodeling due to its distinctive watershed vascularity. Located at the terminal limits of the anterior tibial artery perforators, the pretibial region lacks collateral flow seen in the posterior compartment. Following infection, trauma, or debridement, the lack of underlying muscular bulk leaves relatively avascular structures such as the tibia and extensor tendons without a protective, vascularized cushion to support direct grafting [1].

The recommendation for an exposed tendon stripped of peritenon is vascularized flap coverage. In the pretibial area, even if the peritenon or periosteum is intact, making skin grafting technically possible, it is ill-advised, as even if it manages to heal, it will be chronically thin and subject to breakdown. Large full-thickness defects of the distal third of the leg are typically reconstructed with free flaps due to the lack of reliable local flap options. However, free tissue transfer may not be feasible in medically frail patients with significant cardiac comorbidities, where compromised perfusion and perioperative anesthesia risk limit complex or prolonged reconstructive options [2]. Alternatives to free tissue transfer include serial debridement, negative pressure wound therapy (NPWT), and the use of skin substitutes to establish more reliable vascularized wound cover before split-thickness skin grafting (STSG).

This report describes a staged reconstructive approach utilizing a dermal regeneration template (DRT) in conjunction with NPWT to facilitate neodermis formation over exposed tendon, enabling successful skin grafting in a high-risk patient.

## Case presentation

**Figure 1:**
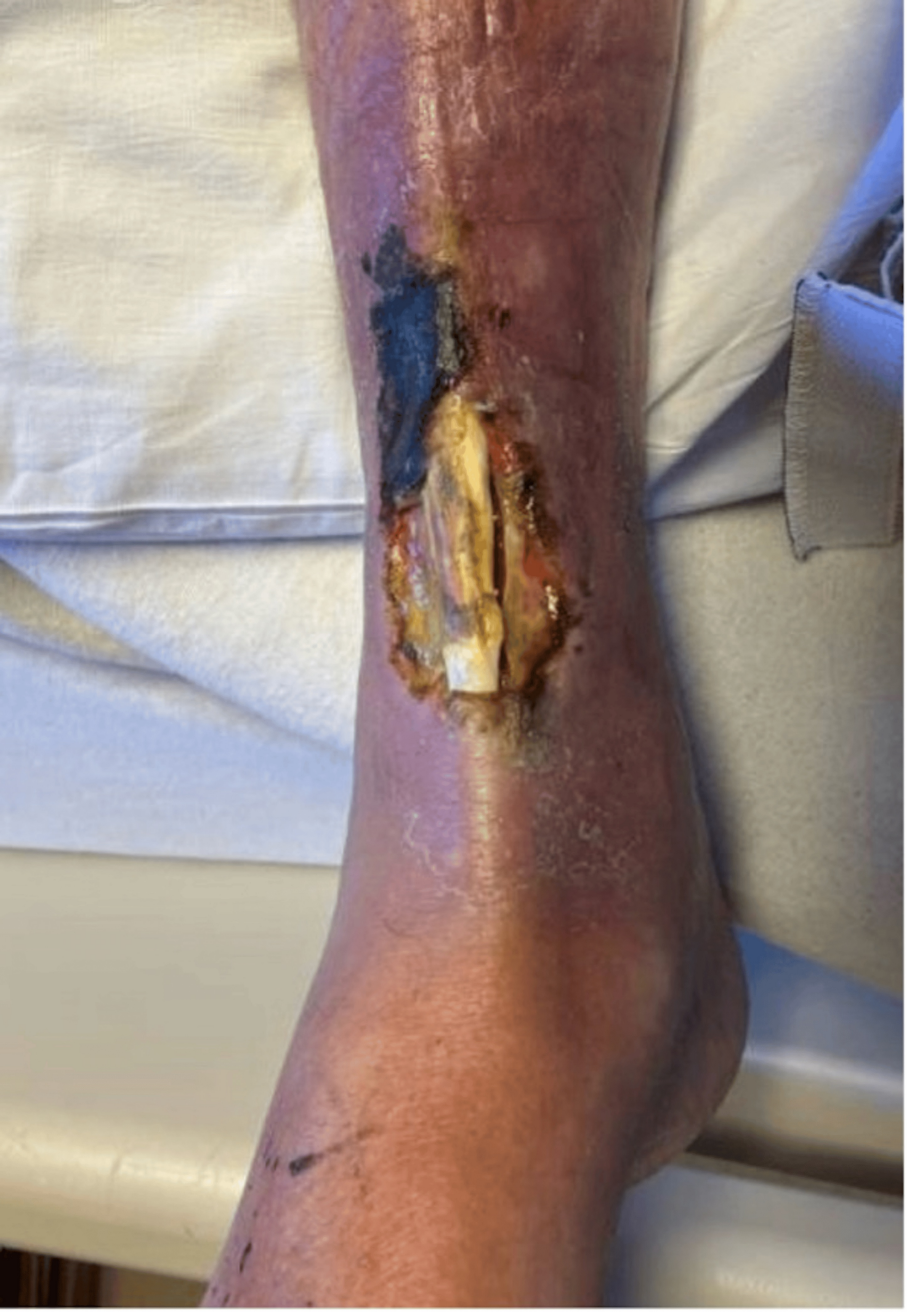
Wound appearance before debridement of the necrotic tissue.

A 74-year-old man with a history of deceased-donor renal transplant on chronic steroids (prednisone 5 mg daily), diabetes mellitus with sequela of neuropathy, peripheral arterial disease, atrial fibrillation on apixaban, and coronary artery disease with previous coronary stenting on aspirin, as well as a remote 30-pack-year smoking history, presented with an 18-week nonhealing right anterior lower-extremity wound. What initially began as a small wound without trauma progressed despite outpatient treatments, resulting in worsening calf claudication and reduced mobility. Etiology was unknown but possibly related to showering of emboli and subsequent tissue necrosis, as the patient had spontaneous dry gangrene of small areas of multiple fingers and toes over the last several months (Figure 1).

**Figure 2:**
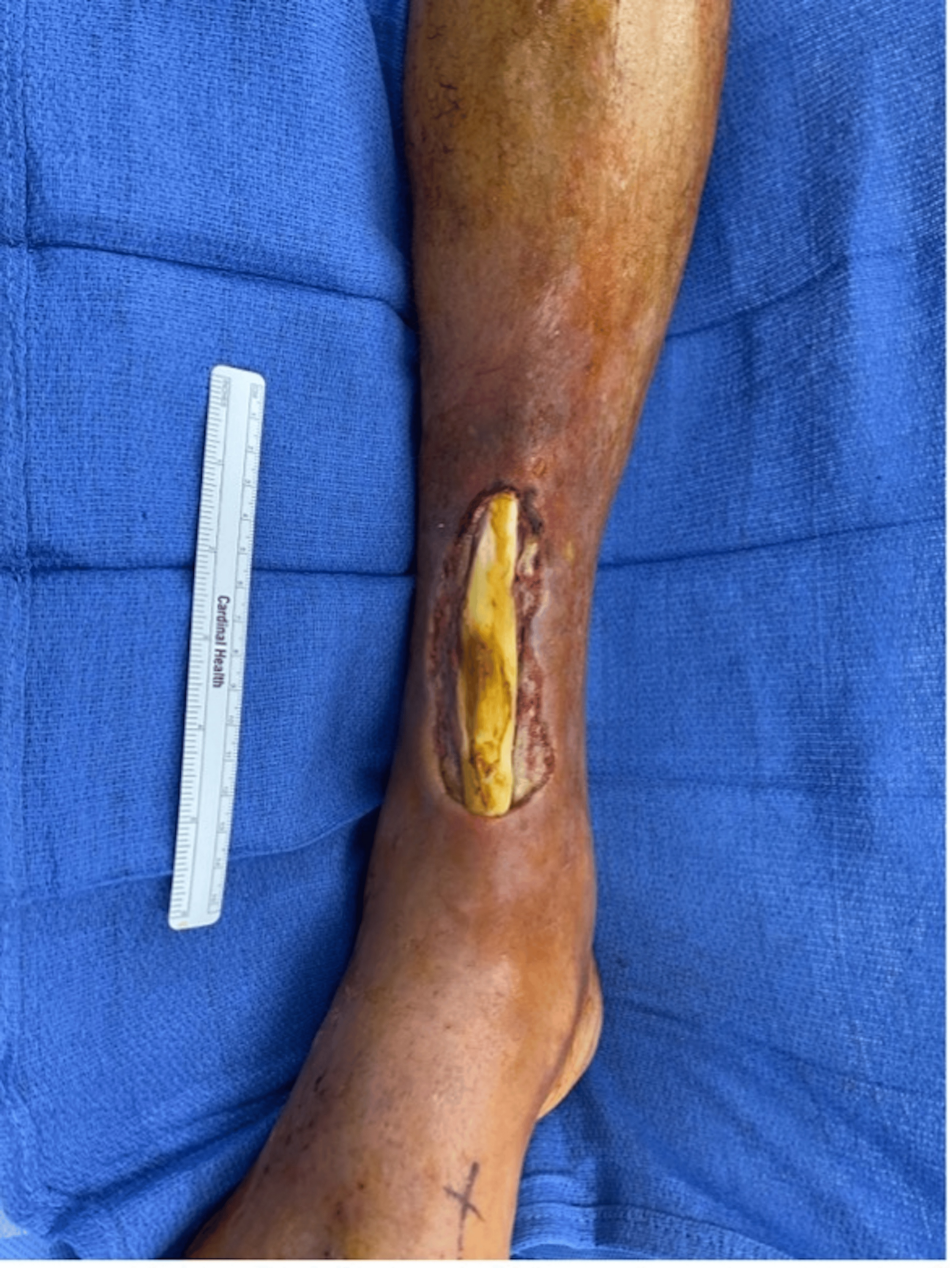
Wound after debridement.

**Figure 3:**
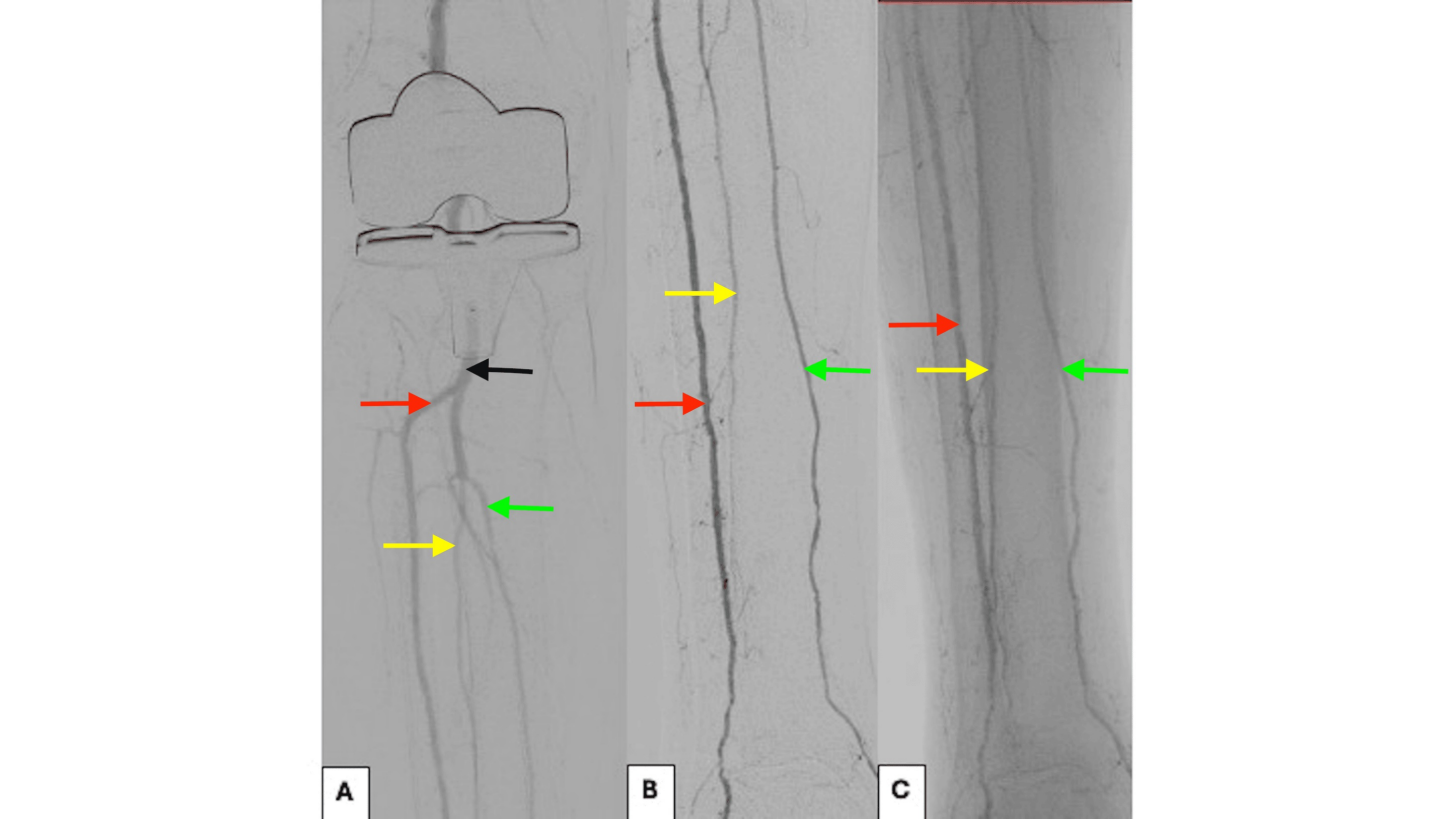
Aortogram with right lower extremity runoff demonstrating diffuse arterial calcification and preserved three-vessel runoff without focal stenosis. (A) At the level of the knee, demonstrating the popliteal artery (black arrow) with bifurcation into the anterior tibial artery (red arrow) and tibioperoneal trunk with proximal visualization of the posterior tibial (green arrow) and peroneal (yellow arrow) arteries. (B, C) From the knee to the ankle, demonstrating patent anterior tibial (red arrow), posterior tibial (green arrow), and peroneal (yellow arrow) arteries.

Initial examination at the time of plastic surgery consultation revealed a 5 × 4 cm pretibial wound in the distal third of the leg with exposed extensor hallucis longus and probable tibialis anterior tendons, surrounding erythema, with an additional 3 × 2 cm dry eschar. The wound contained fibrinous debris without purulent drainage (Figure 2). The patient had non-palpable but Dopplerable pedal pulses. Imaging demonstrated extensive vascular calcifications and a soft-tissue defect without underlying fracture or osteomyelitis. His ankle-brachial index was noncompressible. Lower-extremity angiography demonstrated patent iliac, femoral, and popliteal arteries with intact three-vessel runoff to the ankle and extensive arterial calcification. There were no distinct areas for revascularization during his angiogram (Figure 3).

**Figure 4:**
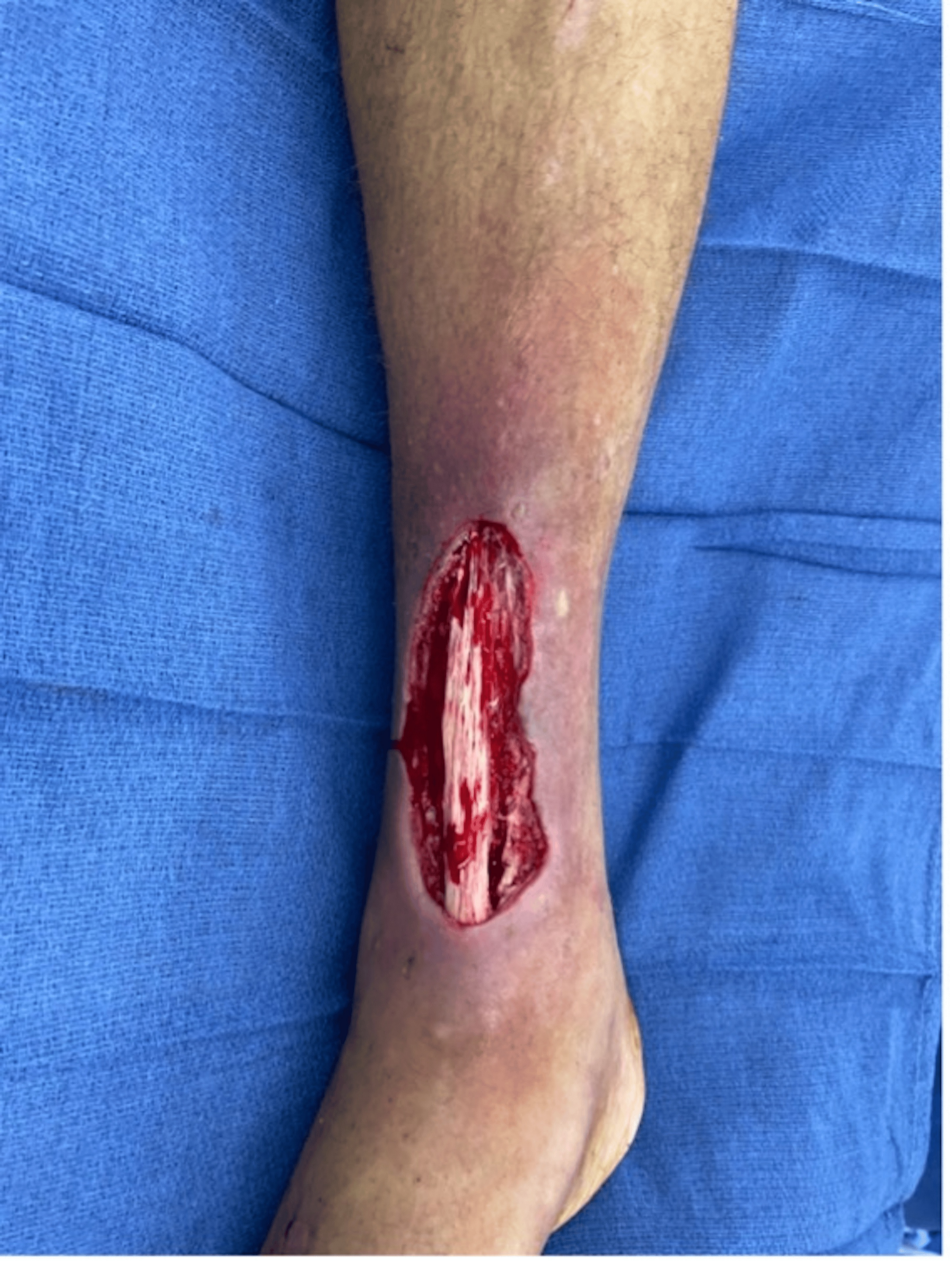
Appearance of wound after debridement just before the application of the dermal regeneration template.

Given his history of renal transplant, systemic atherosclerotic disease, and cardiac history, free tissue transfer was considered high risk. Local myocutaneous flap options were limited due to defect location and poor perfusion, and amputation was only discussed as a last resort. Plastic surgery recommended staged reconstruction, beginning with debridement and coverage with a dermal regeneration template (DRT). The patient underwent operative debridement of devitalized tendon sheath and fibrinous tissue, with the amount of wound exudate noted at approximately 40-50% at the time of the first debridement (Figure 4). 

**Figure 5:**
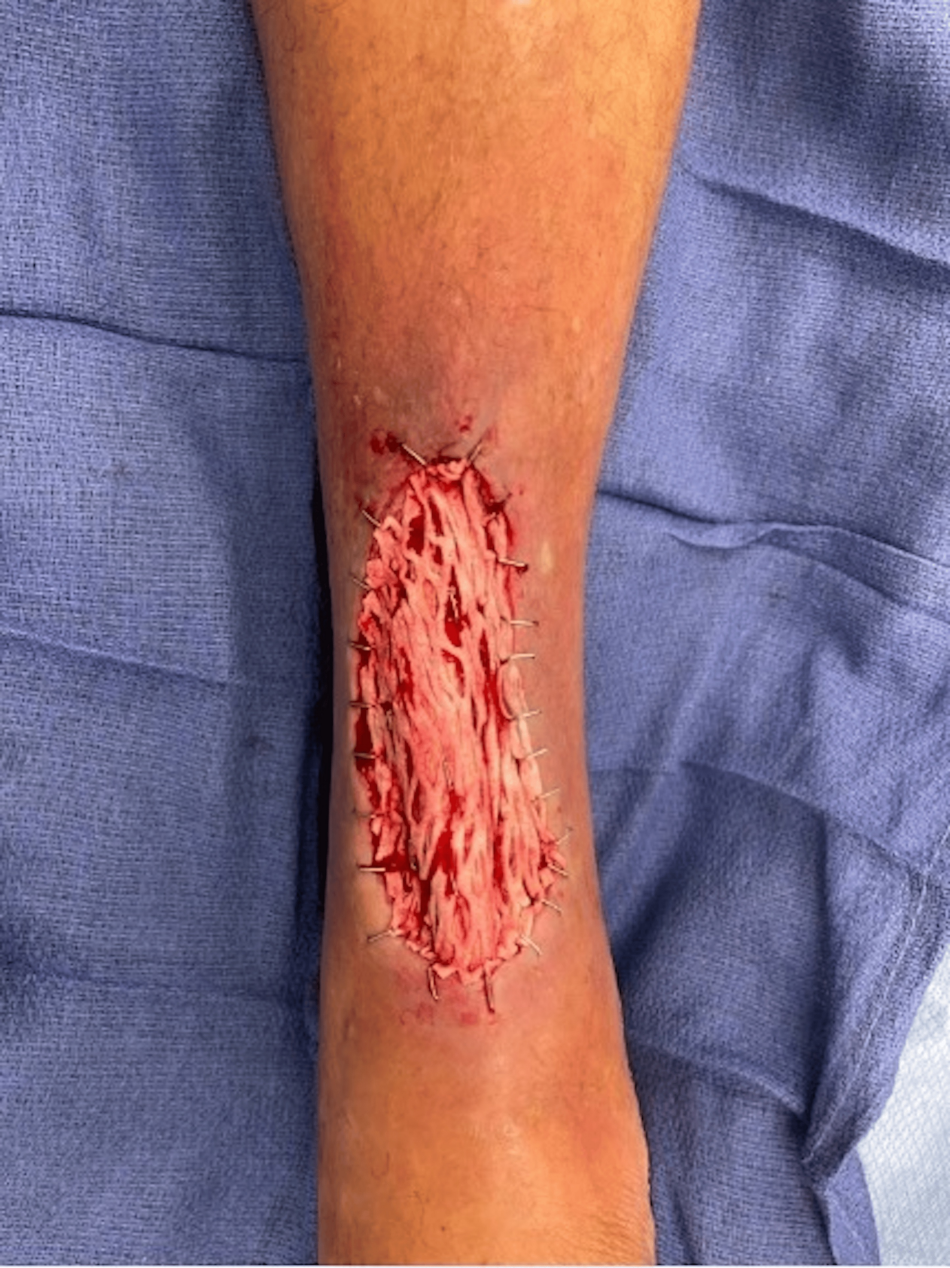
Intraoperative wound appearance following placement of the dermal regeneration template.

**Figure 6:**
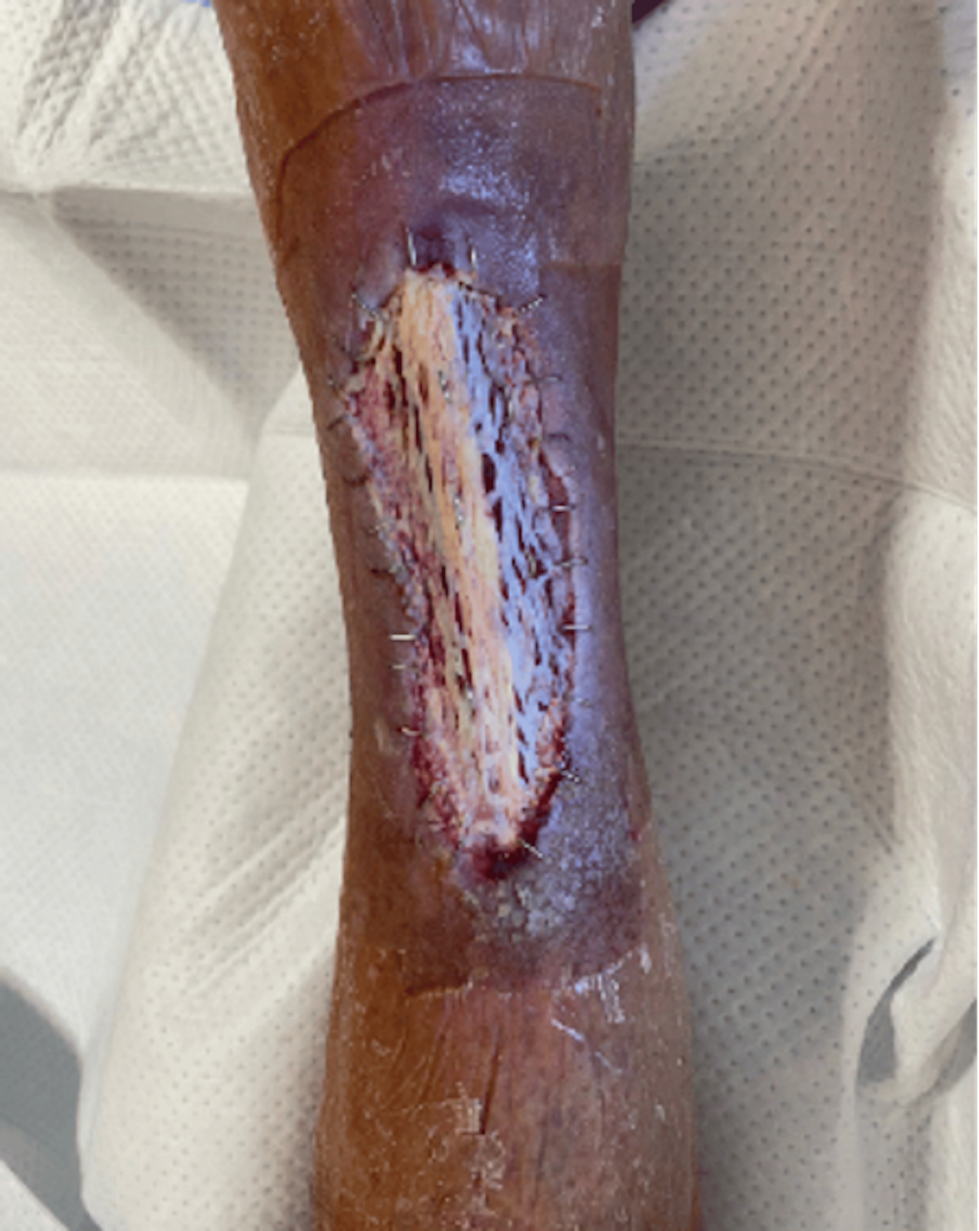
Wound appearance four days following debridement and coverage with the dermal regeneration template, at the first change of the negative pressure wound therapy.

The pretibial wound measured 5 × 12 cm after debridement. A DRT consisting of human allograft decellularized dermis (Somagen®; MTF Biologics, Edison, NJ, USA) was trimmed to size, secured with staples, and supported with a silver-based contact layer followed by NPWT (Figure 5). The extremity was splinted to stabilize the allograft and minimize shear. Early postoperative assessment showed excellent incorporation of the dermal matrix without signs of infection. The patient transitioned to home NPWT changes three times weekly, using black GranuFoam™ dressings (Solventum, Saint Paul, MN, USA) with a silver-based nonadherent dressing covering the wound bed, and weekly wound care follow-ups. He underwent approximately 17 wound vacuum-assisted closure (VAC) from his initial operation to his planned return to the operating room, with the majority completed by home health. With each dressing change, the wound bed was gently cleansed using Vashe® wound solution (Urgo Medical; Fort Worth, TX, USA) and covered with a nonadherent dressing as described above. At his wound care follow-ups, he underwent mechanical debridement four weeks after his initial operation (Figure 6).

**Figure 7:**
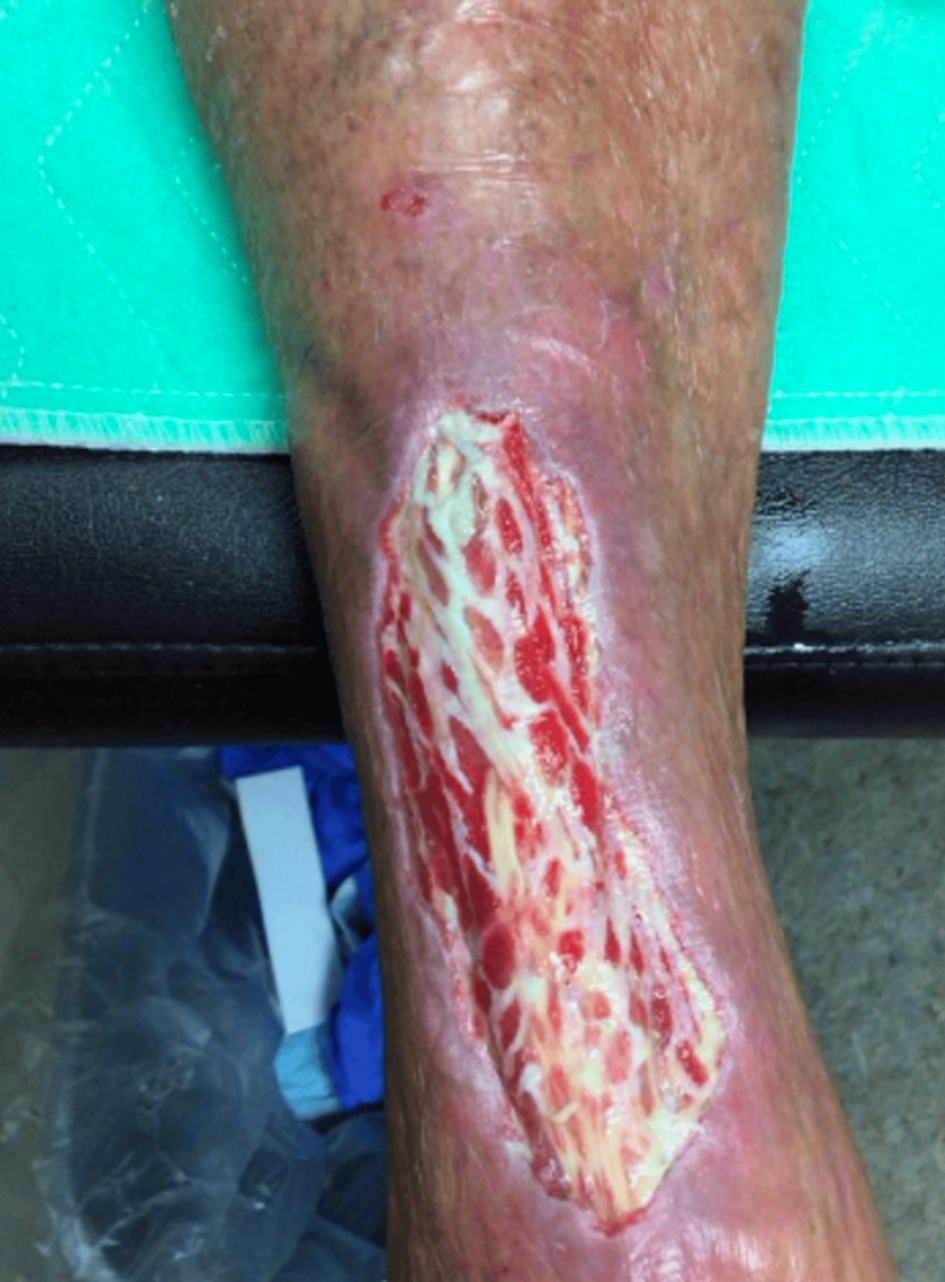
Wound bed showing granulation 24 days after the dermal regeneration template placement.

**Figure 8:**
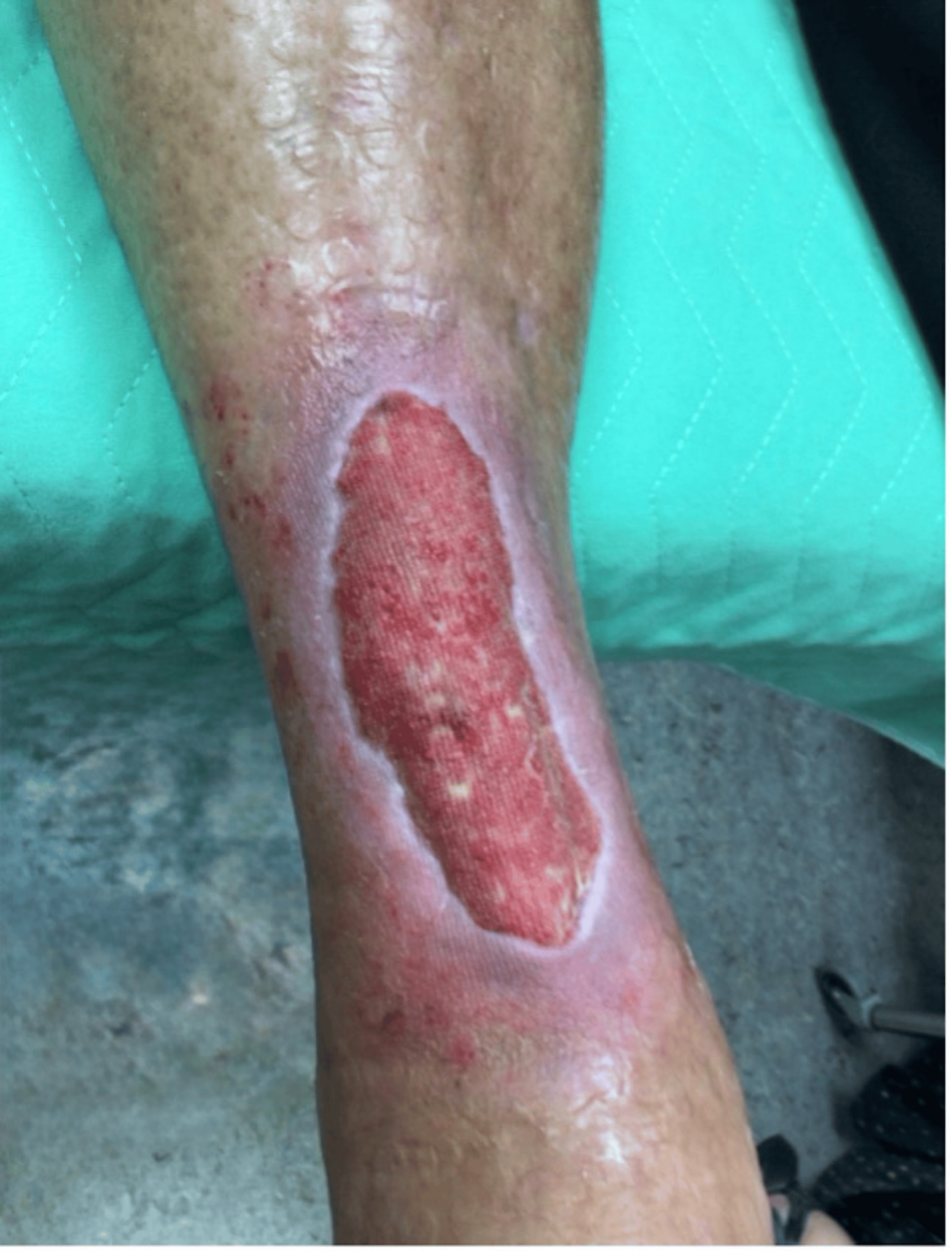
Wound appearance 45 days after dermal regeneration template coverage, demonstrating complete coverage of previously exposed tendon with vascularized granulation tissue.

Over the next four weeks, the wound demonstrated robust granulation (Figure 7), with complete coverage of previously exposed tendon (Figure 8). Given the favorable wound bed maturation, coverage with an STSG was planned. Fifty-five days after the index procedure, the wound bed was prepared, and a 0.014-inch meshed STSG harvested from the ipsilateral thigh was secured over the 11 × 5 cm recipient bed. Negative-pressure wound therapy was applied as a bolster dressing using the V.A.C.® Therapy system (Solventum; Saint Paul, MN, USA), and double Spandagrip® F tubular bandages (Meditech; Canton, MA, USA) were subsequently utilized for compression therapy of the right lower extremity.

**Figure 9:**
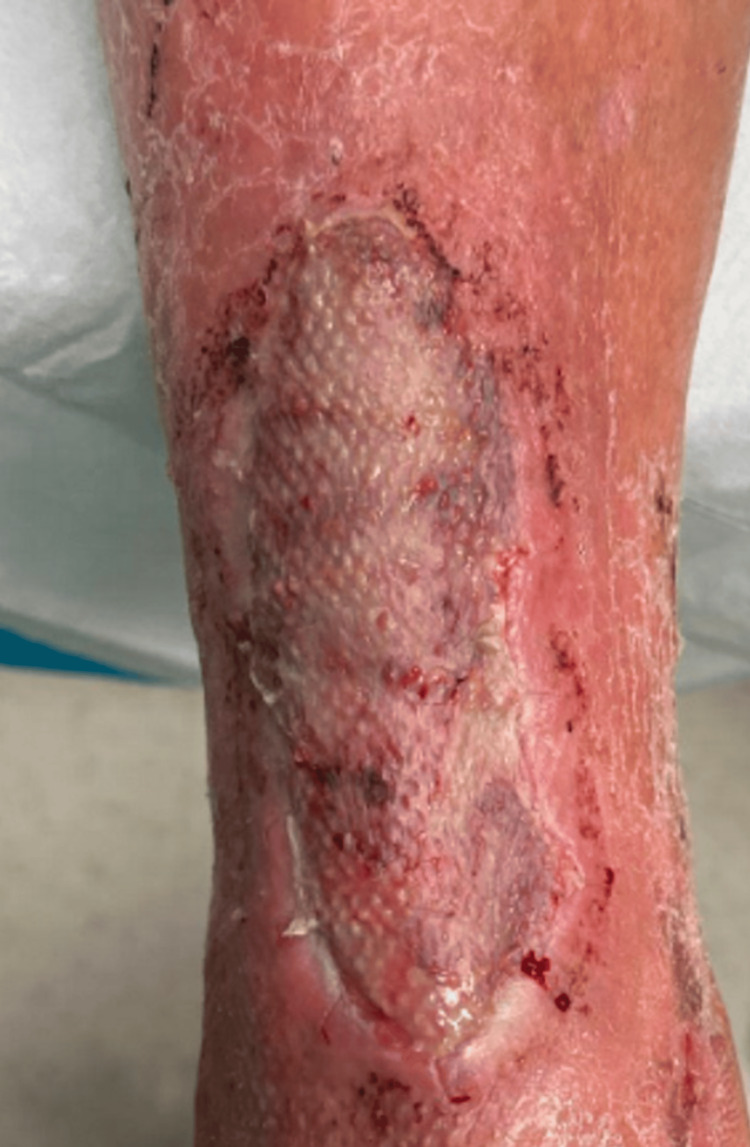
Graft took six days after a split-thickness skin graft procedure.

**Figure 10:**
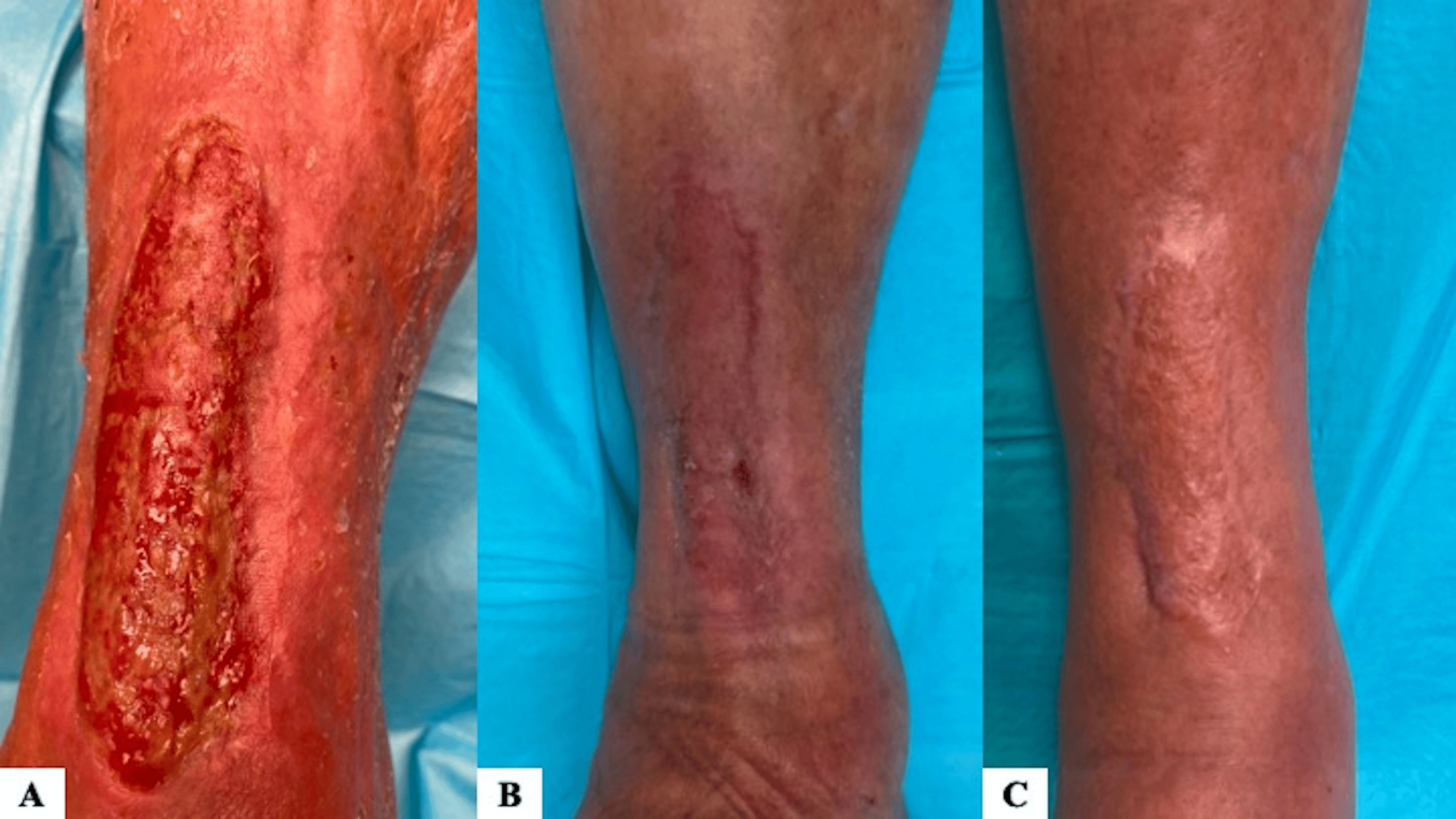
Serial postoperative images demonstrating progressive healing of the split-thickness skin graft (STSG): (A) Well-vascularized graft at three weeks, (B) continued wound healing at two months post-STSG, and (C) completely healed, fully epithelialized graft with stable coverage at five months postoperatively.

At one week postoperatively, the graft demonstrated approximately 98% take (Figure 9). By three weeks, the graft was well vascularized, and the donor site had healed. At two months, the patient had regained full ambulation with only a pinpoint superficial wound remaining. At five months, the wound was fully healed, and somewhat surprisingly, the ankle range of motion demonstrated tendon excursion under the graft (Figure 10). He had returned to his pre-injury functional status and remained highly satisfied with the outcome.

## Discussion

The surgical approach for lower extremity reconstruction in the distal third of the leg with exposed tendon typically requires a microvascular free flap [3]. However, the patient’s past medical history left this "traditional" approach impractical, prompting a transition to a staged reconstructive limb salvage strategy [4]. Recent retrospective data show that utilizing DRTs significantly reduces graft failure (5.2% vs. 19.2%) and facilitates early postoperative mobilization in medically compromised patients by creating a neo-dermis over a traditionally non-adherent surface [5]. 

The success of this reconstruction relied on the bioactive properties of the modern synthetic matrices (such as polylactic acid or biodegradable temporizing matrices) that act as signaling scaffolds, which induce a pro-angiogenic microenvironment [6]. As described in our procedure, the use of a negative pressure wound therapy (NPWT) with the Vacuum-Assisted Closure (VAC) system was essential in converting an avascular tendon surface into a vascularized neo-dermis capable of supporting STSG. NPWT has been shown to increase graft uptake in a complex wound by reducing interstitial edema and promoting mechanical micro-strain, which facilitates effective incorporation of the matrix in the wound bed even in the setting of limited perfusion [7,8].

The use of advanced skin substitutes, including DRTs, does incur significant cost. Due to highly stringent donor criteria and proprietary tissue processing to sterilize and decellularize the tissue, human-derived acellular dermal matrices in particular are quite expensive. Before advising the widespread use of these technologies, one should ensure that their use is either cost-effective or at least comparable to microvascular free tissue transfer. Based on previous analysis and modeling by Kozak et al., the total cost of DRT combined with NPWT and subsequent skin grafting can be significantly lower than that of free flap microsurgical procedures. The main cost of staged reconstruction includes upfront material costs, two operative stage costs, NPWT for a minimum of 13 days, short hospital stays (two days), and outpatient management, which results in approximately $36,000-$38,000. In contrast, a free flap procedure involves a single, but lengthy and more complex operation that makes up the bulk of the cost, along with longer hospital stays (7-10 days), potential intensive care unit (ICU) stays, and a higher potential for post-acute care needs, bringing the average total cost to $53,492. From a cost-effectiveness standpoint, DRT with NPWT staged reconstruction may be more advantageous for defects under 100 cm^2^, as DRT cost directly increases with wound surface area [9]. If the wound is larger or requires multiple stages of skin substitute/DRT application, these cost comparisons may then no longer apply. With large wounds or increased defect complexity, including exposed bone or hardware, free flap reconstruction with native tissue remains superior despite the higher cost, as skin substitutes are not sufficient to meet the demands of the defect for these scenarios [10,11]. Importantly, failure of either method of reconstruction will incur additional treatment costs. Therefore, proper patient selection and surgical decision-making are essential to design a reconstructive plan with the highest chance of success. 

While not well-studied, there is biological plausibility that DRTs may be beneficial to patients with significant co-morbidities, including transplant recipients on immunosuppressive therapies. These patients exhibit persistently impaired wound healing, in which chronic immunosuppression dampens inflammatory signaling required to initiate tissue remodeling and attenuates vascular endothelial growth factor (VEGF)-mediated angiogenesis, contributing to graft failure and postoperative complications [12,13]. When coupled with severe peripheral arterial disease, the microvascular flap transfer becomes a high-risk option due to poor peripheral perfusion and extended operative time, further elevating anesthesia-related risk in medically complex patients.

This clinical reality necessitates a shift in reconstructive strategy: in high-risk cohorts, "engineering" a wound bed through dermal templates is a more reliable alternative to "transplanting" one. Although this approach increases the duration of an exposed wound and requires vigilant infection control with antimicrobial NPWT, it avoids the physiological stress, costs, and altered peripheral perfusion required with microsurgery. As the population of complex transplant recipients grows, careful patient selection and operative planning with consideration for staged approaches will contribute to successful outcomes.

## Conclusions

In high-risk patients with immunocompromised status or poor peripheral perfusion, traditional flap-based reconstructions have lower rates of success or might fail. This case demonstrates that staged strategies, combining DRTs with NPWT, can effectively optimize a vascularized wound bed to support graft integration. Such approaches require careful patient selection and operative planning, providing a reliable pathway to functional and sustainable outcomes in challenging reconstructive scenarios.
